# Melioidosis in Traveler from Africa to Spain

**DOI:** 10.3201/eid1910.121785

**Published:** 2013-10

**Authors:** María I. Morosini, Carmen Quereda, Horacio Gil, Pedro Anda, María Núñez-Murga, Rafael Cantón, Rogelio López-Vélez

**Affiliations:** Author affiliations: Ramón y Cajal University Hospital, Madrid, Spain (M.I. Morosini, C. Quereda, R. Cantón, R. López-Vélez); and National Center for Microbiology, Madrid (H. Gil, P. Anda)

**Keywords:** melioidosis, traveler, Africa, bacteria, Burkholderia pseudomallei, travel, global health, gram-negative, Spain, travel-related infections

## Abstract

The worldwide epidemiology of melioidosis is changing. We describe a case of acute melioidosis in Spain in a patient who had traveled to Africa. A novel sequence type of *Burkholderia pseudomallei* was identified in this patient. Clinicians should be aware of the possibility of melioidosis in travelers returning from melioidosis-nonendemic regions.

Melioidosis is caused by infection with the gram-negative bacterium Burkholderia pseudomallei. Autochthonous cases of melioidosis occur throughout Southeast Asia and northern Australia, but sporadic cases have occurred in other locations, and evidence suggests that the epidemiology of this disease is changing. Cases have been reported in tropical regions of South America, particularly northeastern Brazil (*1*), and other cases have been imported from the Caribbean (*2*). In addition, several cases have been identified in Africa and in travelers returning to European countries from Africa (Table). We report a case of acute melioidosis in a traveler from Africa who was admitted to a hospital in Madrid, Spain.

**Table:** Characteristics of reported cases of human melioidosis with African origin*

Patient age, y/sex	Area visited/ diagnosed	Sample type	Final treatment	Outcome	Clinical risk factors	Possible source	Method of identification†	Reference
64/M	Kenya/Denmark	Blood, urine, sputum	TET+SXT	Recovered	Pulmonary tuberculosis	NR	Serology	(*3*)
12/F	Sierre Leone/The Gambia/	Bony abscesses	CMP+TET	No follow-up	NR	NR	Serology	(*4*)
40/F	Mauritius	Blood	CTX+MDZ	Died	SLE	Rainy season	API 20NE	(*5*)
58/M	Madagascar/La Réunion (France)	Sputum	IMI	Recovered	NR	Unknown	API 20NE, serology, PCR	(*6*)
29/M	The Gambia, Guinea Bissau, Senegal]/Spain	Soft tissue abscesses	CAZ+SXT	Recovered	Hepatitis Diabetes	Rainy season	Vitek II System, API 20NE, PCR	(*7*)
46/F	Nigeria/United Kingdom	Blood	MER	No follow-up	Diabetes	Trip to Nigeria	Chromatography, PCR	(*8*)
60/M	NR/France	Blood	IMI+CIP	Recovered	NR	Unknown	NR	(*9*)
35/F	Madagascar, West Africa/Spain	Blood	CAZ+DOX	Recovered	None	Goat raw milk ingestion	WIDER System, API 20NE, serology, PCR, MLST	This study

*TET, tetracycline; SXT, cotrimoxazole; NR, not reported; CMP, chloramphenicol; CTX, cefotaxime; MDZ, metronidazole; SLE, systemic lupus erythematosus; IMI, imipenem; CAZ, ceftazidime; MER, meropenem; CIP, ciprofloxacin; DOX, doxycycline; MLST, multilocus sequence typing. †API 20NE, bioMérieux, Marcy l’Étoile, France; Vitek II System, bioMérieux; WIDER system (Fco. Soria-Melguizo, Madrid, Spain).

## The Case

A 35-year-old previously healthy woman was admitted to the emergency unit of Ramón y Cajal University Hospital, Madrid, Spain, on March 25, 2011, because of a 4-day fever and arthralgias. She had just returned from an 11-month leisure travel trip through Africa, during which she visited Madagascar and 14 countries in West Africa. At admission, she had fever (39°C) and inflammatory signs on the left ankle. HIV test results were negative, and a chest radiograph showed no abnormalities. The patient was treated with intravenous ceftriaxone (2 g/d); oral doxycycline (100 mg 2×/d) was added the next day.

On the third day, acute progressive dyspnea, cough, hypoxemia, and hypotension developed. A chest and abdominal computed tomography scan showed bilateral lung miliary infiltrates. Bronchoscopy showed bronchial inflammatory signs; mucopurulent secretions were concomitantly observed. Cultures of blood samples taken in the emergency unit were negative, but a gram-negative bacillus was cultured from blood taken on the third day of hospitalization. Gram stain of respiratory specimens taken at bronchoscopy showed a substantial number of polymorphonuclear leukocytes with predominant gram-positive flora. After 48-h incubation at 35 ± 2°C in sheep blood (ambient air) and chocolate agar (5% CO_2_), mostly α-hemolytic colonies of streptococci with a few colonies of saprophytic *Neisseria* spp. were cultured. Treatment was changed to intravenous ceftazidime (2 g 4×/d) and oral doxycycline (100 mg 2×/d), and the patient showed rapid clinical improvement. After 3 weeks of treatment, the patient was discharged with maintenance treatment of oral amoxicillin/clavulanic acid (1 g 3×/d) and doxycycline (100 mg 2×/d), later changed to oral cotrimoxazole (1,920 mg/d) for 3 months course. After a year, the patient had no sequelae or relapsing symptoms attributable to melioidosis.

The aerobic bottles of the 2 sets of blood specimens collected from the patient after hospitalization were positive for gram-negative rods with clear, bipolar staining. Subcultures on sheep blood agar plates (35 ± 2°C) yielded a bacillus forming greyish colonies with an intense, earthy odor. The organism was identified as *B. pseudomallei* with a 79.0% probability score by the WIDER system (Fco. Soria-Melguizo, Madrid, Spain); the API 20NE system (bioMérieux, Marcy l’Étoile, France) identified the isolate as *B. pseudomallei* with the numeric profile 1156577 (99.9% certainty). Mass spectrometry (MALDI-TOF MS; Bruker Daltonics, GmbH, Leipzig, Germany) initially identified the isolate as *Burkholderia thailandensis*, likely because of the paucity of entries in the database at that time (*10*).

The full 16S rRNA gene (≈1,500 bp) of the isolate was amplified and sequenced. Nucleotide sequence alignment results were identical to results of a *B. pseudomallei* isolate from GenBank (accession no. AF93059). Susceptibility testing was performed (WIDER system), and results were interpreted according to Clinical and Laboratory Standards Institute *Burkholderia cepacia* guidelines (*11*). The isolate was susceptible (MIC, µg/mL) to ceftazidime (<1), meropenem (<2), minocycline (<2), levofloxacin (<2), and cotrimoxazole (<2/38).

The isolate was sent to the National Reference Center for Microbiology (Majadahonda, Spain), where it was labeled BpSp2 and analyzed by a reverse line blot PCR that enables the specific identification of *B. mallei*, *B. pseudomallei*, and *B. thailandensis* (*7*). The isolate was further characterized by multilocus sequence typing (MLST) (*12*); the allele sequence and profile identified were submitted to the public database (http://bpseudomallei.mlst.net) for number assignation, and the obtained sequence type (ST) was compared with other published STs. In addition, the concatenated sequences of the 7-MLST genes (3,399-bp) of the BpSp2 strain and the STs available in the public database were aligned by using ClustalX (www.clustal.org), and neighbor-joining phylogenetic trees were constructed by using MEGA4 software (*13*). *B. pseudomallei* strain NCTC 10276 was used to prepare immunofluorescence assay slides. Two serum samples from melioidosis patients in Thailand were used as positive controls, and a serum sample from a patient in Spain who had bacteremia caused by *B. cepacia* was used as a negative control. A convalescent-phase serum sample from the patient, a sample from her husband, and serial serum samples from 7 hospital workers suspected of potential exposure were analyzed.

The BpSp2 isolate was confirmed as *B. pseudomallei* by PCR by using the specific probe for this pathogen (data not shown). MLST showed a new allelic profile for this strain, including a new allele for *ndh* that was numbered 39. This ST was identified as 879 (1, 1, 10, 2, 6, 1, 39) in the database and is close to the human ST349 (Martinique and Spain) (*7*), ST7 (Vietnam), and ST662 (France). These STs, however, showed 2 different loci compared with ST879, which grouped in the same clade with ST662 and ST7 but also with ST26 (Niger) and ST707 (Nigeria) (Figure). Other than ST29, ST20, and ST319 (Burkina Faso, Niger, and Mauritius, respectively), the isolates from Africa grouped in the same clade with the STs from South America and the Caribbean (Figure).

**Figure:**
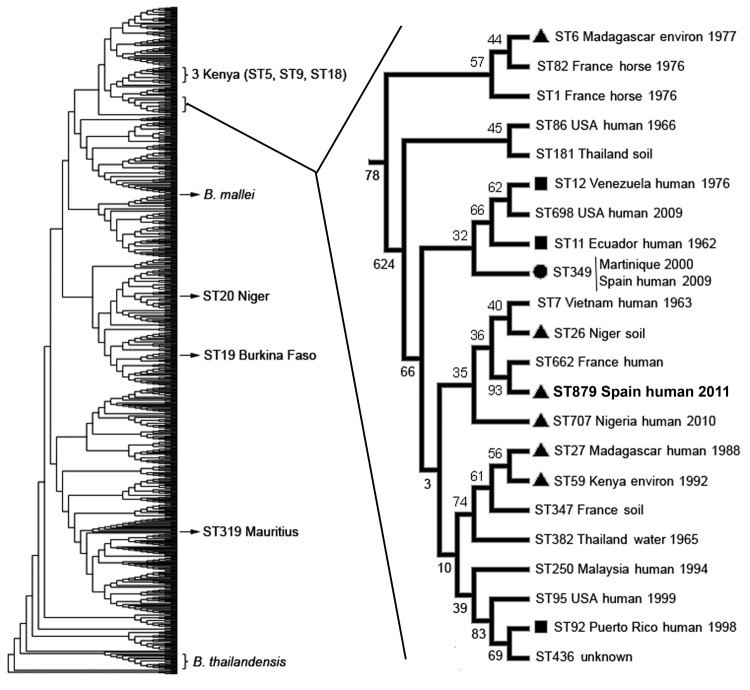
Phylogenetic position of *Burkholderia pseudomallei* isolate BpSp2, sequence type (ST) 879 (**boldface**), from a patient in Spain who had traveled to Africa. The dendrogram was built by using 852 isolates from the public *B. pseudomallei* database (http://bpseudomallei.mlst.net). The clade in which most STs from Africa, South America, and the Caribbean are located has been enlarged; location, source type, and year collected are indicated for each isolate. Black circle indicates isolates from Africa; black squares indicate isolates from South America or the Caribbean; black triangles indicate ST349 from Martinique and the first patient described in Spain (*7*). Location of other STs from Africa, *B. mallei*, and *B. thailandensis* are also indicated. Environ, environment.

The patient’s serum showed an IgG titer of 640; IgM was negative. The patient’s husband and the laboratory workers were negative for IgG and IgM, except that in 1 of the workers, a titer of 40 for IgM was detected, but no seroconversion occurred.

## Conclusions

Although Africa is not considered a melioidosis-endemic zone, melioidosis in humans has been reported; melioidosis was confirmed in a traveler returning from Africa to Spain in 2009 (*7*). For the patient we report, common predisposing factors for melioidosis were not observed, and an inoculation event or definite exposure to contaminated soil or water were not identified. The patient had consumed raw, nonpasteurized goat milk, and *B. pseudomallei* has been isolated from this food (*14*), but this possible link was not conclusive.

Confirmatory diagnosis of *B. pseudomallei* infection is isolation of the pathogen from clinical samples of patients, but the identification of *B. pseudomallei* can be elusive because automated systems used in clinical microbiology laboratories may misidentify it as *B. cepacia* (*15*). However, API 20NE has proven to give accurate identification, as has mass spectrometry with an extensive database support. Despite high variability among the *B. pseudomallei* isolates, most of the STs from Africa grouped in the same clade and seemed to be phylogenetically related, particularly with strains from South America and the Caribbean. The phylogenetic analysis strongly suggests that BpSp2 has an African origin.

In conclusion, because of difficulties in diagnosis, cases of melioidosis outside areas to which it is endemic may be more common than realized. An increase in nongovernment organization and medical cooperation programs in Africa, as well as leisure travelers moving to and from Africa, may have resulted in increased frequency of some infections, such as imported melioidosis. These findings require clinicians to recognize the disease and microbiologists to identify the causative agent.
